# Impact of therapist change after initial contact and traumatic burden on dropout in a naturalistic sample of inpatients with borderline pathology receiving dialectical behavior therapy

**DOI:** 10.1186/s40479-017-0067-3

**Published:** 2017-06-22

**Authors:** Carolin Steuwe, Michaela Berg, Martin Driessen, Thomas Beblo

**Affiliations:** 1 Research Department, Clinic of Psychiatry and Psychotherapy, Ev. Kliníkum Bethel, Bielefeld, Germany; 2 Department of Psychology, Clinical Psychology and Psychotherapy, Bielefeld University, Bielefeld, Germany

**Keywords:** Borderline personality disorder, Posttraumatic stress disorder, Dialectical behavior therapy, Dropout

## Abstract

**Background:**

This study focused on the predictors of therapy dropout in a naturalistic sample of patients with borderline pathology receiving dialectical behavior therapy (DBT) in an inpatient setting. We assumed that the change of the therapist between DBT-briefing and start of DBT-treatment as well as comorbid posttraumatic stress disorder (PTSD) and childhood trauma history were associated with elevated dropout.

**Methods:**

Eighty-nine participants with borderline pathology (≥ 3 borderline personality disorder criteria) receiving an inpatient DBT program completed a quality assurance questionnaire set assessing demographic information and pretreatment psychopathology during the days of their inpatient stay. Beyond that, changes of therapists were documented. The predictor analyses were investigated with generalized estimating equations.

**Results:**

The dropout rate was 24.7%. A change of therapist between DBT-briefing and treatment as well as high childhood emotional abuse was associated with premature termination of treatment. Higher values of physical neglect during childhood were associated with a protective effect on treatment dropout. Surprisingly, this was also true for comorbid PTSD.

**Conclusions:**

This study supports the importance of therapy process variables as predictors of therapy dropout in borderline pathology. A change of therapist between DBT-briefing and treatment was associated with an increased vulnerability for dropping out of treatment and should therefore be avoided if possible. Against our hypotheses, a comorbid PTSD was even protective with regard to DBT dropout. Therefore, this severely suffering patient group should not be rejected from treatment assuming them to be too unstable for psychotherapy. However, results need to be replicated. ClinicalTrials.gov Identifier: NCT03018639, retrospectively registered on January 9, 2017.

## Background

Borderline personality disorder (BPD) is associated with an elevated treatment dropout rate irrespective of the therapeutic approach [3, 5, 10, 39]. Dialectical behavior therapy (DBT) has most often been proven to be an effective treatment for patients with borderline pathology [26]. However, even for DBT, a mean dropout rate of 27.3% was found in a meta-analysis [26]. Despite the prevalence and high clinical relevance, the attention drawn to this phenomenon has increased only in the last years. Samples in different therapeutic settings and therapeutic approaches have mostly been examined with respect to “patient variables” such as demographic and clinical characteristics.

Only a few studies have investigated the predictive value of therapeutic processes on treatment completion in psychotherapy for BPD [5]. An important process factor is the therapeutic alliance, that can be defined as the overall bond between therapist and patient evolving during the process of therapy [24]. Sharf et al. [36] found an effect size of Cohen’s d = .55 to describe the association between the therapeutic alliance and therapy dropout in adult individual therapy across all mental disorders. Interpersonal difficulties represent core difficulties in patients with BPD and may affect the therapeutic alliance [1]. Patients show frantic efforts to avoid real or imagined abandonment and a pattern of unstable and intense interpersonal relationships characterized by alternating between extremes of idealization and devaluation. Therefore, a predictive value of the therapeutic alliance on treatment retention rates seems specifically likely in this patient group. However, results are heterogeneous. Whilst most studies found that a poor therapeutic alliance predicts dropout in BPD [5, 39, 40], a recent study of Barnicot et al. [4] showed that frequent use of skills in DBT was even more important than therapeutic alliance regarding treatment retention rates. A recent study investigating treatment characteristics and first-session relationship variables as predictors of dropout in traumatized youth, found that the level of approval of therapy after the first-session significantly influenced treatment completion rates [33]. We assume that a change of therapist after the first therapeutic contact may be experienced as a rupture in the therapeutic process that hypothetically harms the therapeutic alliance and thus influences treatment dropout.

Furthermore, comorbid disorders, such as a generally higher burden of axis I disorders [39], alcohol and substance abuse, and anorexia nervosa, have been found to be associated with dropout [27, 28]. It is known that patients with BPD and comorbid posttraumatic stress disorder (PTSD) are a particularly burdened population with an increased level of general distress, elevated numbers of suicide attempts and non-suicidal self-injury [21, 34]. DBT is increasingly recommended for PTSD, however, patients with BPD and comorbid PTSD benefit less from DBT as compared to patients with BPD alone [22, 41]. Accordingly, patients with BPD and comorbid PTSD are affected by many of the risk factors associated with dropout described above and may therefore be at risk for dropping out of treatment. Nevertheless, the relationship between comorbid PTSD and therapy dropout is poorly understood. Arntz et al. [3] found that childhood physical abuse predicted treatment discontinuation. To the best of our knowledge, there is no study targeting the effect of a comorbid PTSD on (DBT) dropout rates.

Results are also heterogeneous in terms of the impact demographic factors and clinical characteristics. Younger age has been associated with higher dropout rates [27, 29]. However, other studies did not find evidence for an influence of demographic factors [5]. In a recent study, Landes et al. [29] found an increased pretreatment level of general distress to be a significant predictor of dropout, whereas other studies did not find an association between dropout and pretreatment symptom load [8, 35].

To date there are no studies investigating the effect of a therapist change on treatment process of BPD. The purpose of this study was to investigate factors related to dropout from inpatient treatment in a large sample of patients with Borderline pathology. More specific, we hypothesized that (i) the change of the therapist between DBT-briefing and start of DBT-treatment as well as (ii) comorbid PTSD and childhood trauma history are associated with elevated dropout. In addition, demographic and clinical factors cited above that were reported to predict dropout in adult populations, should be controlled for.

## Methods

### Recruitment and assessment procedures

For this study, all patients aged from 18 to 65, discharged from our personality disorder inpatient unit between December 2012 and August 2016, and fulfilling three or more criteria for BPD as defined by DSM-IV (Borderline Personality characteristics; BPC) were considered (*n* = 89). Exclusion criteria included inability to contract and consent, other severe mental disorders (bipolar disorder, acute psychosis), an inability or unwillingness to avoid alcohol, illicit or not prescribed drug use during the inpatient stay, simultaneous participation in other treatment studies, pregnancy or breastfeeding, an inability to negotiate a non-suicide agreement, ongoing traumatic contact with the perpetrator, and a Body Mass Index <16.5. We also excluded patients with a treatment history on our ward that may have confounded the impact of the initial contact with a therapist in the DBT-briefing.

### Procedure and measures

Within the first week of their inpatient stay each participant, who proved to be positive for the inclusion criteria were informed of the aims and conditions of participation. Participants gave their written informed consent to study participation and publication of results. Ethical standards were in accordance with the declaration of Helsinki. Afterwards each participant completed a quality assurance questionnaire set. Besides demographic information, the set contains the Borderline Symptom List (BSL; [11]) to assess BPD symptom severity [12], the Beck Depression Inventory II (BDI-II; [23]) to assess depressive symptoms, the Posttraumatic Stress Diagnostic Scale (PDS; [16]) to assess PTSD symptom severity as well as the Symptom-Checklist assessing the psychopathologic burden (SCL-90-R; [15]). Here we only report the Global Severity Index (GSI). The Childhood Trauma Questionnaire (CTQ; D. [6]) assessed the types of traumatic experiences that had occurred within the family context. Pathological dissociation was assessed via the *Fragebogen zu dissoziativen Symptomen* (FDS; [38]), the German version of the Dissociative Experiences Scale (DES; E. M. [7]), and quality of life as evaluated via the World Health Organization – Quality of Life Questionnaire (WHO-QOL; [2]).

### Treatment

The treatment in our inpatient personality disorder unit is certified by the German DBT Board of Certification (DDBT; consecutively certified since 2007, last certification: 22.03.2016). As common in DBT settings, patients were seen in outpatient consultations before DBT starts (DBT-briefing). The briefing includes examination of the patient, assessment of the treatment history, indication for treatment, assessment of inclusion and exclusion criteria for treatment. It lasted one hour. As often as possible, the therapist who conducted the briefing also undertook the treatment, usually two to three months later. However, for organizational reasons this was not always feasible; in these cases a different therapist took over after DBT-briefing (documented as therapist change) between DBT briefing and treatment. A change of therapist was in no case caused by clinical considerations. There were no additional contacts after DBT-briefing and DBT-treatment. There was no change of therapist during treatment for any reason (organizational or clinical). The length of the inpatient stay was eight to twelve weeks, within the sixth week the discharge date was fixed depending on the patients’ progress, aims, and needs.

#### Dialectical behavior therapy

DBT is a cognitive-behavioral treatment program that was developed to treat suicidal patients with BPD [30]. Over a period of eight to twelve weeks, participants received weekly 50-min sessions of individual treatment (ten sessions over the ten weeks) plus weekly group treatments as follows: 180 min of skills training (24–30 sessions over the ten weeks), 45 min focusing on mindfulness and psychoeducation on BPD (8–10 sessions over the ten weeks). The program’s purpose is to help patients achieve the following therapeutic goals: (1) reduction of suicidal behaviors, (2) reduction of therapy-interfering behaviors, and (3) other risky or destabilizing behaviors. Standard DBT aims to achieve these goals by (1) conveying behavioral capabilities (skills), (2) motivation for applying these skills, (3) generalization of learned skills to the patient’s natural environment, (4) structuring the treatment environment to reinforce functional behavior, and (5) conveying therapeutic resources and motivation to effectively treat patients with BPD.

#### Standard inpatient care

SIC includes all non-specific therapeutic elements. Over a period of eight to ten weeks, participants received twice-weekly 30-min sessions of supportive talks with the primary nurse, twice-weekly sessions of art- or music therapy, and weekly sessions of body therapy. Beyond that, all patients receive morning rounds, movement therapy, and learned relaxation techniques. Patients also receive usual psychopharmacological treatment that is documented.

### Definition of dropout

Treatment dropout was assessed by recording whether the participant was discharged from our ward earlier than week eight or earlier than the final discharge date fixed in week six. Reasons for drop-out were documented (on part of the patient vs. on part of the ward). Contingency management was the reason for discharge on part of the ward in all cases. It includes positive consequences for functional behavior and negative consequences for dysfunctional behavior. Dysfunctional behavior was defined as suicidal behavior, non-suicidal self-injury, drug use, and therapy disturbing behavior (missing sessions, violating common ward rules). In case dysfunctional behavior was shown repeatedly (usually four times), a patient was discharged from treatment.

### Data analyses

The initial analyses included group comparisons with independent-sample t-tests and χ^2^ statistics as well as explorative correlation analyses. Because of the nested nature of the data (patients nested within therapists), the predictor analyses were investigated with generalized estimating equations (GEE; [19]). Data were complete with regard to the scales of interest. To account for missing data (one missing value concerning the DES) in non-targeted descriptive variables, we performed multiple imputations. We used SPSS imputations (multiple imputations) to impute 50 values for the missing observation.

## Results

### Sample characteristics and dropout

The sample consisted of 89 treatment-seeking patients with borderline pathology. Participants had an average age of 29.8 years (*SD* = 9.95), 76.4% were female (*n* = 68). 23.9% of participants were currently living in a relationship and they reported an average of 10.6 years of basic school education (*SD* = 1.48). 84.3% (*n* = 75) of participants fulfilled the diagnostic criteria of BPD, 15.7% (*n* = 14) showed borderline characteristics only (BPC; 3–4 BPD-criteria). There were no significant differences in demographic characteristics or pretreatment symptom severity between patients meeting full criteria of BPD and patients showing BPC. Participants reported an average of 2.9 different types of (recurring) lifetime traumas (*SD* = 1.78, *range* = 1–7) as indicated by the PDS event checklist. For the CTQ, childhood maltreatment means are listed as follows: emotional abuse, *M* = 15.9 (*SD* = 5.37, *cut-off* = 10, above cut-off: 82%), physical abuse, *M* = 9.55 (*SD* = 4.50, *cut-off* = 8, above cut-off: 38%), sexual abuse *M* = 8.71 (*SD* = 5.59, *cut-off* = 8, above cut-off: 28%), emotional neglect, *M* = 17.62 (*SD* = 4.56, *cut-off* = 15, above cut-off: 63%), and physical neglect, *M* = 10.54 (*SD* = 4.08, *cut-off* = 8, above cut-off: 48%). Participants met criteria for an average of 1.2 current Axis I disorders including PTSD (*SD* = 0.99) and 0.9 Axis II disorders in addition to BPD (*SD* = 0.32).

In this treatment-seeking sample of patients with borderline personality characteristics treated in an inpatient setting, the dropout rate was 24.7% (*n* = 22). Of these, 50% (*n* = 11, 12.4% of the total sample) were discharged on part of the ward, 41% (*n* = 9, 10.1% of the total sample) were discharged on their own requests, 9% (*n* = 2, 2.2% of the total sample) ended therapy because of other reasons.

Detailed information of the circumstances that led to dropouts is depicted in Table 1. 52.8% (*n* = 47) of patients experienced a change of therapist between DBT-briefing and treatment. The mean duration of the inpatient stay was 41.73 days (*SD* = 21.04, *range* = 3–71) for the dropout group and 66.48 days (*SD* = 13.63, *range* = 34–104) for the completer group. Comparisons of the dropout vs. completer group showed that both groups did not differ with regard to demographic characteristics and pretreatment symptom severity except for childhood physical neglect with higher values in the completer group. The dropout group, however, experienced a therapist change between DBT-briefing and treatment significantly more often (see Table 2).

**Table 1:** Reasons for DBT dropout (case by case)

	Discharge on part of the ward (*n* = 11)	Discharge on patient’s request (*n* = 9)	Other (*n* = 2)
Reasons for dropout	1. Suicidality 2. Repeated alcohol consumption 3. Aggressive behavior 4. Repeated extensive eating attacks 5. Repeated alcohol consumption and violation of other rules for our ward 6. Insult of fellow patients and team members 7. Physical attack towards fellow patient 8. Repeated therapy-interfering behavior (violating rules, missing sessions) 9. Started a love affair with a fellow patient 10. Theft and damage to property 11. Physical attack towards fellow patient	1. Ambivalence with regard to therapy 2. Felt uncomfortable in inpatient setting 3. Non-suicidal self-injury and difficulties opening in therapy sessions 4. Drug use at home, rejected to continue treatment 5. Felt uncomfortable in inpatient setting 6. Did not return to the clinic after family difficulties at home 7. Felt wronged by team members 8. Disappointment by the treatment and anger at team members 9. Felt wronged by team members and fellow patients	1. Repeated medical and social problems 2. Transfer to neurosurgical clinic

**Table 2:** Demographics and diagnostic information of subgroups and group differences

	Group				Statistic	
Characteristic	Completers (*n* = 67)		Non-Completers (*n* = 22)			
	*Mean*	*SD*	*Mean*	*SD*	*t*	*p*
Age (years)	29.24	10.00	31.32	9.85	−.849	.398
School (years)	10.65	1.50	10.45	1.44	.553	.582
Number of axis-I-disorders	1.16	1.07	1.09	0.75	.298	.766
	*N*	*%*	*N*	*%*	*χ* ^*2*^	*p*
Sex (female)	49	73.13	19	86.36	1.608	.205
Current BPD	55	82.09	20	90.91	.972	.324
Current PTSD	50	74.63	13	59.09	1.933	.164
Current alcohol/substance abuse	11	16.40	4	18.18	.216	.898
Change of therapist	28	41.79	19	86.36	13.203	>.001*
	*Mean*	*SD*	*Mean*	*SD*	*t*	*p*
Global Severity Index (SCL-90-R)	1.33	0.59	1.28	0.58	.371	.711
Borderline Symptom List	162.53	60.90	151.87	73.45	.682	.497
Beck Depression Inventory	29.28	11.96	26.08	12.25	1.082	.282
Dissociative Experiences Scale	21.62	12.78	22.01	13.62	−.122	.903
Number of traumatic event types (PDS)	3.10	1.76	2.59	1.82	1.179	.242
Childhood Maltreatment (CTQ)						
Emotional abuse	15.67	5.36	16.68	5.44	−.764	.447
Physical abuse	9.83	5.15	8.68	4.50	.937	.351
Sexual abuse	9.09	5.58	7.55	5.60	1.125	.264
Emotional neglect	17.93	4.50	16.68	4.70	1.112	.269
Physical neglect	11.04	4.20	9.00	3.34	2.075	.041*

*BPD* Borderline Personality Disorder, *PTSD* Posttraumatic Stress Disorder, *SCL* Symptom Checklist, *PDS* Posttraumatic Diagnostic Scale, *CTQ* Childhood Trauma Questionnaire. Statistic: *df* = 1 for *χ* ^*2*^-tests and *df* = 87 for *t*-tests

*p ≤ .05

### Predictors of dropout

Explorative correlation analyses between treatment dropout and applied measures revealed a significant association only between change of therapist and dropout (*r* = .385, *p* < .001). All other measures (e.g. subscales of SCL-90-R [impulsivity, anxiety]) did not correlate with treatment dropout.

The separate relationship between each variable of interest and dropout is presented in Table 3. Findings show that comorbid PTSD, childhood emotional abuse, childhood physical neglect and therapist change are significantly associated with dropout status whereas other variables are not. Results revealed a protective effect against dropout by comorbid PTSD (dropout rate in PTSD group: 20.6%, dropout rate in non-PTSD-group: 34.6%) and childhood physical neglect. Higher values in emotional abuse as well as a change of therapist (dropout rate in group with change: 40.4%, dropout rate in group without change: 7.1%) were associated with an increased risk for dropping out of treatment. For further results see Table 3. The working correlation matrix in GEE did not indicate that any effect traces back to a specific therapist or a combination of therapists in briefings or treatments. Multiple imputations revealed comparable results for all analyses.

**Table 3:** Generalized estimating equation predicting dropout

Predictors	*Est* (*se*)	*p*	*OR*	*OR 95% CI*
Age	−2.91 (.64)	.083	1.42	0.96–2.12
Substance use	0.55 (.71)	.440	1.73	0.43–7.01
PTSD	1.18 (.52)	.024*	0.31	0.11–0.86
Axis I disorders	0.05 (.26)	.850	1.05	0.63–1.76
GSI	−0.14 (.25)	.591	0.87	0.53–1.43
CTQ-EA	1.00 (.35)	.004*	2.72	1.38–5.38
CTQ-PA	−0.36 (.44)	.421	0.70	0.30–1.67
CTQ-SA	−0.03 (.32)	.934	0.98	0.53–1.80
CTQ-EN	−0.10 (.28)	.724	0.91	0.53–1.56
CTQ-PN	−0.76 (.38)	.047*	0.47	0.22–0.99
Change of Therapist	1.52 (.38)	<.001*	4.59	2.19–9.59

*Est* Estimate, *se* standard error, *OR* odds ratio, *CI* confidence interval, *PTSD* Posttraumatic Stress Disorder, *GSI* Global Severity Index, *CTQ* Childhood Trauma Questionnaire, *EA* Emotional Abuse, *PA* Physical Abuse, *SA* sexual abuse, *EN* Emotional Neglect, *PN* Physical Neglect

*p ≤ .05

## Discussion

This study investigated baseline variables and one therapeutic process variable related to dropout from 10-week inpatient DBT among patients with borderline pathology. Our hypotheses were partially confirmed. High childhood emotional abuse was associated with premature termination of treatment. However, higher CTQ-values of physical neglect during childhood were associated with a protective effect on treatment dropout. This was also true for comorbid PTSD. In addition, a change of therapist between DBT-briefing and treatment was associated with a significantly elevated risk of drop-out.

The dropout rate (24.7%) found in this naturalistic sample of treatment seeking patients with borderline pathology is comparable to dropout rates found in previous inpatient-DBT-studies [10, 25, 26, 35]. Reasons for dropout in around half of all cases was discharge on the patients’ own request (e.g. because of ambivalence with regard to the treatment, conflicts with fellow patients); the other half was discharged on part of the staff because of repeated dysfunctional behaviors (e.g. substance use, aggressive behaviors, negative therapy-interfering behaviors). In sum, reasons for dropout were balanced out. No patient explicitly mentioned a change of therapist being a reason for dropout.

This study supports the importance of therapy process variables as predictors of therapy dropout in borderline pathology. Patients that experienced a change of therapist between DBT-briefing and treatment were more vulnerable for dropping out of treatment. In the dropout-group twice as much patients experienced a change of therapist as compared to the completer group. Assuming that a change of therapist is a burden for the therapeutic alliance, the results of this study are in line with studies finding that a poor patient or therapist-rated alliance can predict dropout [37]. The early point in time of therapist change may be important. The briefing is used to gain an agreement on tasks and goals accompanied by empathetic resonance. Patients who agree to these tasks/goals and feel comfortable in the therapeutic bond within the very first session will decide to participate in the DBT program. Despite the fact that patients are explained that a change of therapist is probable, patients might nevertheless be affected through therapist change in many ways. For example, patients might be disappointed or frightened by the idea that goals or agreements reached in the DBT-briefing are not valid anymore or that the therapeutic bond is not the same. Patients with borderline pathology experience relationships in extremes of idealization and devaluation [1]. The therapeutic alliance that might be idealized within or in the aftermath of the DBT-briefing might switch to devaluation of the therapeutic alliance or of the whole treatment by a change of therapist. Trust in the treatment and commitment to change, which have also been shown to be associated with dropout [5], might decrease. The importance of the first session relationship variables for dropout later on in treatment has been shown in traumatized youth [33], also suggesting that the therapeutic alliance is a particularly important component of therapy in individuals with histories of childhood abuse [14].

In line with Arntz et al. [3], we found childhood abuse to be predictive of dropout in DBT. In our study, emotional abuse was predictive of premature therapy termination which was by trend significant in Arntz et al. [3]. Childhood physical abuse, unlike in the latter study, was not significantly associated with dropout. Sexual and non-sexual abuse can cause a wide range of mental health consequences [32]. Particularly emotional abuse is related to emotional regulation and interpersonal difficulties in adulthood, moderated by maladaptive schemas such as mistrust, abandonment, and shame [13, 31], that may complicate treatment and therefore lead to an elevated dropout risk. Therefore, our findings may be moderated by emotion regulation deficits (specifically non-acceptance of emotions) that have recently been found to predict DBT dropout rates [29]. As an inpatient setting is highly accommodative, physical neglect might have a protective effect on dropout. Basic needs (e.g. food, medical care) that might have been deprived during childhood, are fulfilled during the inpatient stay, thereby increasing the likelihood of therapy completion.

Comorbid PTSD, against our prediction, was not associated with elevated dropout rates. Indeed, a comorbid PTSD was even protective with regard to DBT dropout. It is known that patients with BPD and PTSD benefit less from DBT [20]. This is, however, not solely due to premature dismissal from treatment (dropout rate in the comorbid PTSD subgroup: 20.6%). Patients were committed to the treatment and they were not discharged more frequently on part of the ward for repeatedly showing dysfunctional behaviors, despite of their likely increased symptom load. This finding encourages treating patients with BPD and PTSD with specialized treatments for both disorders. Our hypothesis that an elevated general symptom load which is elevated in patients with BPD and PTSD in comparison to BPD patients without this comorbidity [22] would lead to elevated dropout rate was not confirmed, even though the PTSD patients significantly differed from the patients without PTSD in terms of general symptom severity (GSI). Opposed to the findings of Landes et al. [29], but in line with previous studies [8, 35] we did not find an effect of the general symptom severity predicting dropout rates. The protective effect of PTSD might be due to an elevated psychological strain; however, we also did not find a protective effect of GSI. Irrespective of their elevated general symptom severity or emotion regulation deficits, our study suggests that patients with comorbid PTSD have the motivation and/or competence to maintain an inpatient treatment. Suffering from PTSD on the one hand and the hope to be helped may also be the background of our findings. Therefore, this severely suffering patient group should not be rejected from treatment assuming them to be too unstable for psychotherapy. However, this result needs to be replicated.

### Limitations

The naturalistic setting of this study involves several limitations that are worth noting. Patients did not receive structured diagnostic interviews. The diagnosis of PTSD was assured only by a self-reporting questionnaire. The Posttraumatic Stress Diagnostic Scale is used in a wide range of clinical and research contexts and is known to entail a high degree of confidence (Foa et al. [18] found 82% agreement between diagnosis using the PDS and the Structured Clinical Interview for DSM (SCID-I; [17])). However, further studies should include structured diagnostic interviews for PTSD such as the Clinician Administered PTSD Scale [9]. The high prevalence of PTSD diagnosis in our sample may be due to our sample being highly burdened; however, it may also be an overestimation of prevalence by the PDS. Thus, our findings cannot be generalized to outpatient DBT. It is also likely that the low comorbidity rate reported for the study sample underestimates the true comorbidity because the diagnosis of comorbid disorders was only based on clinical judgment indicated in the discharge report. Again, a valid comorbidity rate should be ensured by using structured clinical interviews.

Furthermore, future studies should assess to what degree and at what time a change of therapist is, as hypothesized in this study, a rupture in therapeutic alliance. The therapeutic alliance should be directly and much more intensively assessed by self-report-ratings of therapists and patients. Also we were not able to cover all variables that have been shown to be predictive of DBT dropout in previous studies, such as emotion regulation skills. Future studies should include both trauma history as well as emotion regulation variables.

### Implications

In the past, many studies assessing reasons for dropout in patients with BPD have focused on demographic variables and symptom severity to predict dropout rates. This study illustrates the importance of further assessing therapy process variables and the therapeutic alliance. Unlike patient variables, therapy process variables can mostly be influenced by the clinic, therapist or provider. Therapist changes should be avoided in the treatment of BPD patients and if that is inevitable, knowing that a change of therapist increases risk for dropout, the therapist is able to pick up this risk, e.g. by putting special attention on engagement, working alliance, and commitment strategies. Therapists may anticipate difficulties and repair a potential rupture in the therapeutic alliance. The finding that more childhood emotional abuse is associated with premature dropout may implicate to consider pretreatment distress with measures like the CTQ. Putting additional attention on engagement, working alliance, and commitment strategies as well as improving emotion regulation strategies for those with increased scores may be important implications for treatment. Finally, regarding patients with comorbid PTSD, clinicians may be encouraged to challenge these patients to the therapy they are committed to (e.g. DBT, trauma therapy), despite the high pretreatment symptom severity.

## Conclusions

Based on the results of the current study, patients with borderline pathology who experienced a change of therapist between DBT-briefing and treatment and childhood emotional abuse treatment are more likely to dropout from DBT. Additional research is needed to replicate these results in other – including outpatient - samples. Especially, it is necessary to better understand the therapy process variables involved, such as structural therapeutic elements of the therapeutic setting as well as the course of the therapeutic alliance. Furthermore, research should include other variables that may affect dropout (satisfaction with treatment, expectations of treatment etc.).

## Abbreviations

BDI-II: Beck depression inventory II
BPC: Borderline personality characteristics
BPD: Borderline personality disorder
BSL: Borderline symptom list
CAPS: Clinician Administered PTSD Scale
CTQ: Childhood trauma questionnaire
DBT: Dialectical behavior therapy
DES: Dissociative experiences scale
DSM: Diagnostic and statistical manual of mental disorders
FDS: Fragebogen zu dissoziativen symptomen
GEE: Generalized estimating equations
GSI: Global severity index (SCL-90-R)
PDS: Posttraumatic stress diagnostic scale
PTSD: Posttraumatic stress disorder
SKID-I: Structured clinical interview for DSM-IV-TR axis-I-disorders
SCL-90-R: Symptom-Checklist 90 items, revised version
WHOQOL: World Health Organization – Quality of Life Questionnaire
